# The effect of dimethyl sulfoxide on the induction of DNA strand breaks in plasmid DNA and colony formation of PC Cl3 mammalian cells by alpha-, beta-, and Auger electron emitters ^223^Ra, ^188^Re, and ^99m^Tc

**DOI:** 10.1186/s13550-016-0203-x

**Published:** 2016-06-03

**Authors:** Roswitha Runge, Liane Oehme, Jörg Kotzerke, Robert Freudenberg

**Affiliations:** Department of Nuclear Medicine, University Hospital/Faculty of Medicine Carl Gustav Carus, Technische Universität Dresden, Fetscherstrasse 74, D-01307 Dresden, Germany

**Keywords:** α-emitter, LET, Plasmid DNA, Cellular survival, DMSO

## Abstract

**Background:**

DNA damage occurs as a consequence of both direct and indirect effects of ionizing radiation. The severity of DNA damage depends on the physical characteristics of the radiation quality, e.g., the linear energy transfer (LET). There are still contrary findings regarding direct or indirect interactions of high-LET emitters with DNA. Our aim is to determine DNA damage and the effect on cellular survival induced by ^223^Ra compared to ^188^Re and ^99m^Tc modulated by the radical scavenger dimethyl sulfoxide (DMSO).

**Methods:**

Radioactive solutions of ^223^Ra, ^188^Re, or ^99m^Tc were added to either plasmid DNA or to PC Cl3 cells in the absence or presence of DMSO. Following irradiation, single strand breaks (SSB) and double strand breaks (DSB) in plasmid DNA were analyzed by gel electrophoresis. To determine the radiosensitivity of the rat thyroid cell line (PC Cl3), survival curves were performed using the colony formation assay.

**Results:**

Exposure to 120 Gy of ^223^Ra, ^188^Re, or ^99m^Tc leads to maximal yields of SSB (80 %) in plasmid DNA. Irradiation with 540 Gy ^223^Ra and 500 Gy ^188^Re or ^99m^Tc induced 40, 28, and 64 % linear plasmid conformations, respectively. DMSO prevented the SSB and DSB in a similar way for all radionuclides. However, with the α-emitter ^223^Ra, a low level of DSB could not be prevented by DMSO. Irradiation of PC Cl3 cells with ^223^Ra, ^188^Re, and ^99m^Tc pre-incubated with DMSO revealed enhanced survival fractions (SF) in comparison to treatment without DMSO. Protection factors (PF) were calculated using the fitted survival curves. These factors are 1.23 ± 0.04, 1.20 ± 0.19, and 1.34 ± 0.05 for ^223^Ra, ^188^Re, and ^99m^Tc, respectively.

**Conclusions:**

For ^223^Ra, as well as for ^188^Re and ^99m^Tc, dose-dependent radiation effects were found applicable for plasmid DNA and PC Cl3 cells. The radioprotection by DMSO was in the same range for high- and low-LET emitter. Overall, the results indicate the contribution of mainly indirect radiation effects for each of the radionuclides regarding DNA damage and cell survival. In summary, our findings may contribute to fundamental knowledge about the α-particle induced DNA damage.

**Electronic supplementary material:**

The online version of this article (doi:10.1186/s13550-016-0203-x) contains supplementary material, which is available to authorized users.

## Background

The molecular nature of DNA damage is characterized by the physical properties of energy deposition and the chemical environment. The dependence on these parameters can be evaluated using simple experimental systems that allow the quantification of DNA damage. For this purpose, plasmid molecules are frequently used to study the yields of single strand breaks (SSB) and double strand breaks (DSB) induced by α-particles and heavy ion beams [1–5]. Direct and indirect irradiation effects can be differentiated by means of DMSO which prevents radical-mediated, indirect DNA damage [4, 6, 7].

As underlined by several studies, the severity of the DNA damage depends on the linear energy transfer (LET) of the radiation quality [8–10]. Generally, DNA damage in plasmid DNA and cells by α-particles is assumed to be only partly protectable by DMSO due to the decreased role of the free radical processes [2, 9, 11].

A variety of studies were undertaken to find out whether high-LET emitters such as α-particles and Auger electrons interact with DNA by direct effects or rather by indirect effects. However, the published results regarding this question are inconsistent. Direct effects are held for the primary mechanism for α-particles and Auger electron emitters in mammalian cells [12, 13]. In contrast, other authors indicated that DMSO has a protective effect against the cellular inactivation by Auger electron emitters [12–15].

Recent studies relating to direct effects from DNA incorporated ^125^I-molecules in plasmids have shown: If ^125^I has a critical distance to the DNA helix, the DSB generation switches from a direct to an indirect mechanism [16].

The growing clinical interest in the use of the α-emitter ^223^Ra to treat bone metastases of prostate cancer [17–20] indicate a need for further basic research because ^224^Ra in ankylosing spondylitis patients demonstrated late effects that were not foreseen [21, 22].

To elucidate the contrary findings regarding the effects from high LET emitters, we studied the DNA damage and reduction of cellular survival induced by α-particles from ^223^Ra in comparison to β-and Auger electron emitters. Thus, we determined the yield of DNA damage in plasmid molecules and clonogenic cell survival in rat thyroid cells exposed to ^223^Ra, ^188^Re, and ^99m^Tc. To identify direct and indirect radiation effects corresponding to the LET of the radionuclides, the radioprotection by DMSO modulation was also examined.

## Methods

### Radionuclides

The α-particle emitter ^223^Ra-radiumdichloride (^223^RaCl_2_, Xofigo) was provided by Bayer Vital GmbH (Leverkusen, Germany) with an activity concentration of 1000 kBq/ml. ^223^Ra (half-life 11.4 days) decays through a cascade of short lived α- and β-particle emitters. During each decays from ^223^Ra, four α-particles are generated resulting in the emission of approximately 28 MeV of energy with 95 % of the energy from the α-emissions [17]. The maximum LET of all alpha particles from ^223^Ra and their decay products is about 250 keV/μm [23, 24].

The β-emitter ^188^Re-perrhenate (^188^ReO_4_^−^) was obtained by elution of a 40-GBq alumina-based ^188^W/^188^Re generator (Isotope Technologies Garching GmbH, Germany). The physical characteristics of ^188^Re are half-life 17 h, maximal β-energy 2.1 MeV, LET 0.19 keV/μm, and γ-emission 155 keV.

^99m^Tc-pertechnetate (^99m^TcO_4_^−^) was eluted from a ^99^Mo/^99m^Tc generator (Ultra-TechnekowTM DTE, Mallinckrodt Medical B. V., Le Petten, The Netherlands) with 0.9 % saline. The radionuclide ^99m^Tc (half-life 6 h) emits γ-rays with energies of 140 keV. Simultaneously with the γ-emission, ^99m^Tc emits 5.1 electrons per decay; 4.8 of these have kinetic energies below 2 keV, and a mean LET that is higher than 10 keV/μm.

### Plasmid DNA

The plasmid pUC19 (2686 base pairs, molar mass 1.75 × 10^6^ Dalton) originating from *E. coli* ER2272 was purchased from New England Biolabs, Ipswich, UK. The DNA stock solutions were diluted in TE buffer (10 mM Tris-HCl and 1 mM EDTA, pH 7.5) to achieve a final concentration of 0.1 μg/μl and stored at −20 °C. Only samples containing >95 % closed circular DNA forms were used. Digestion of pUC19 was performed using the restriction enzyme BamH I (Invitrogen, Karlsruhe, Germany) to obtain linear DNA.

### Irradiation procedure for plasmid DNA

Plasmid samples, each containing 200 ng of plasmid DNA at the same concentration (0.1 μg/μl) and various amounts of radioactive solutions, were placed into micro tubes (Eppendorf, Hamburg, Germany). One radiotracer-free micro tube served as the control. Solutions of ^223^Ra, ^188^Re, or ^99m^Tc were added to the pUC19 plasmids (0.05–1.5 MBq/ml; 17–211 MBq/ml; 530–6615 MBq/ml, respectively) in a total volume of 20 μl. Each sample was incubated for a period of 24 h at room temperature to allow for the accumulation of the radiation dose (^223^Ra, 0–540 Gy; ^188^Re, 0–500 Gy; ^99m^Tc, 0–500 Gy).

The plasmid samples were treated according the following procedure:

- The first set was subjected to various amounts of radioactive solutions of ^223^Ra, ^99m^Tc, and ^188^Re without any modulation.
- The second set was subjected to radioactive solutions of ^223^Ra, ^99m^Tc, and ^188^Re with the radical scavenger DMSO at a final concentration of 0.2 M DMSO.
- The third set was subjected to a constant activity concentration of radionuclides treated with various concentrations of DMSO.

### Measurement of DNA damage in pUC19 plasmids

After irradiation, 10 μl of each irradiated DNA solution was mixed with 1.25 μl of the loading buffer. The mixture was placed into the wells of a 1.4 % agarose gel in TAE buffer. The samples were run at 4 V/cm for 120 min at 6 °C. After electrophoresis, supercoiled (SC), open circular (OC), and linear (L) forms of plasmid DNA were identified based on their mobility differences in the gel. The gel was stained with ethidium bromide (0.5 μg/ml) followed by visualizing the different forms of DNA (Diana III Digital Imaging System, Raytest, Straubenhardt, Germany). The DNA damage was quantified by using the software Fiji.

### Calculation of SSB and DSB

The mean number of single strand breaks *N*_SSB_ and the mean number of double strand breaks *N*_DSB_ per plasmid were calculated based on the theoretical considerations presented by Bishayee et al. [12]. *N*_DSB_ was estimated based on the relative proportion *L* of linear plasmids by

$$ {N}_{\mathrm{DSB}}=L/\left(1-L\right). $$

*N*_SSB_ was calculated using *L* and the fraction *SC* of supercoiled plasmids by

$$ {N}_{\mathrm{SSB}} = \mathrm{L}\mathrm{n}\left(\left(1-L\right)/\mathrm{S}\mathrm{C}\right). $$

### Dosimetry

Dose calculations were performed for plasmid DNA experiments in accordance to the MIRD formalism [25]. In brief, the dose from a source volume to a target volume is calculated as the product of the time integrated activity in the source volume and a source-target-specific *S* value.

As mentioned above, each plasmid DNA sample was irradiated in a total volume of 20 μl in micro tubes. The geometric shape of the radionuclide solution containing the plasmid DNA corresponds to a spherical drop with 1.68 mm radius. *S* values for ^223^Ra, ^99m^Tc, and ^188^Re were estimated by Monte Carlo simulations using Geant4 assuming a homogeneous activity distribution inside the sphere to calculate the mean dose in the sphere. The time integrated activities, corresponding to the total number of decays in the micro tube ${N}_{\mathrm{MT}}$, were calculated by

$$ {N}_{\mathrm{MT}}=\frac{A\cdot {T}_{1/2}}{ \ln (2)}\left(1- \exp \left[- \ln (2)\frac{t_{\mathrm{expo}}}{T_{1/2}}\right]\right), $$

with $A$ being the applied radioactivity, ${T}_{1/2}$ being the radionuclide half-life, and ${t}_{\mathrm{expo}}$ being the exposition time.

### Cell culture

The rat thyroid epithelial cell line PC Cl3 (Clinical Cooperation Unit Nuclear Medicine, DKFZ, Heidelberg, Germany) is a substrain of the Fisher rat thyroid cell line FRTL-5 (ATCC number CRL-8305). Cells were grown in Ham’s F12 medium (Gibco Invitrogen GmbH, Darmstadt, Germany) supplemented with 5 % fetal bovine serum and a cocktail of six hormones as described earlier [26]. The culture medium was renewed every 2–3 days as well as before the application of the radioactivity.

### Irradiation procedure for the PC Cl3 cells

PC Cl3 cells were plated at low cell densities (200–25,000 cells/well) in six-well multititer plates in a volume of 2 ml/well of activity-free standard medium and were precultured for 16 h before the addition of the radionuclides. After a medium change, the ^223^Ra-, ^188^Re-, or ^99m^Tc-radioactive culture medium was added (0.55–8.3 kBq/ml, 0.25–5.0 MBq/ml, and 1.25–25 MBq/ml, respectively) to the cells, and the cells were exposed to the radionuclides for 24 h.

The PC Cl3 cells were irradiated and modulated by DMSO according the experimental setup as described above for the plasmid samples.

Non-irradiated control aliquots were included in all of the experiments. To obtain comparable results, the experiments were carried out using PC Cl3 cells at identical passages.

### Measurement of clonogenic cell survival

Following irradiation, the radioactive supernatant was discarded, and the cells were washed three times with PBS (37 °C). To allow the cells to form colonies, they were maintained in activity-free standard medium at 37 °C in a 5 % CO_2_ atmosphere over a period of 10 days. The colonies were then fixed with ethanol and stained with crystal violet for 30 min. Colonies of more than 50 cells were scored as survivors, and the plating efficiency for each sample was estimated based on the initial number of seeded cells. The clonogenic cell survival was calculated as the relative plating efficiency of the treated versus the untreated samples. Survival data from three independent experiments were fitted by weighted linear regression [27], whereby the applied activity concentration $A$ was taken instead of dose. Formally, this corresponds to a linear-quadratic model $\mathrm{S}\mathrm{F}={e}^{-\left( aA+b{A}^2\right)}$. Both a linear only and a linear and quadratic relation between the natural logarithms of the fraction $\mathrm{S}\mathrm{F}$ and the activity concentration were considered.

To quantify the protective contribution from DMSO, protection factors $\left(\mathrm{P}\mathrm{F}\right)$ were calculated as the ratio of linear parameters $a$ from the regression analysis for irradiation in the presence (${a}_{+\mathrm{DMSO}}$) and absence (${a}_{-\mathrm{DMSO}}$) of 0.2 M DMSO for each radionuclide,

$$ \mathrm{P}\mathrm{F}=\frac{a_{-\mathrm{DMSO}}}{a_{+\mathrm{DMSO}}}. $$

### Statistical analysis

The yields of SSB and DSB in plasmid DNA are presented as mean values and standard error of the mean from three independent experiments. Triplicate samples were prepared for each treatment and experimental condition. Statistical analysis of the survival data from three independent experiments was performed by linear regression (SPSS Statistics 23.0 IBM, Armonk, NY, USA). *P* values of less than 0.05 were considered to be significant.

## Results

### Plasmid DNA

**Fig. 1:**
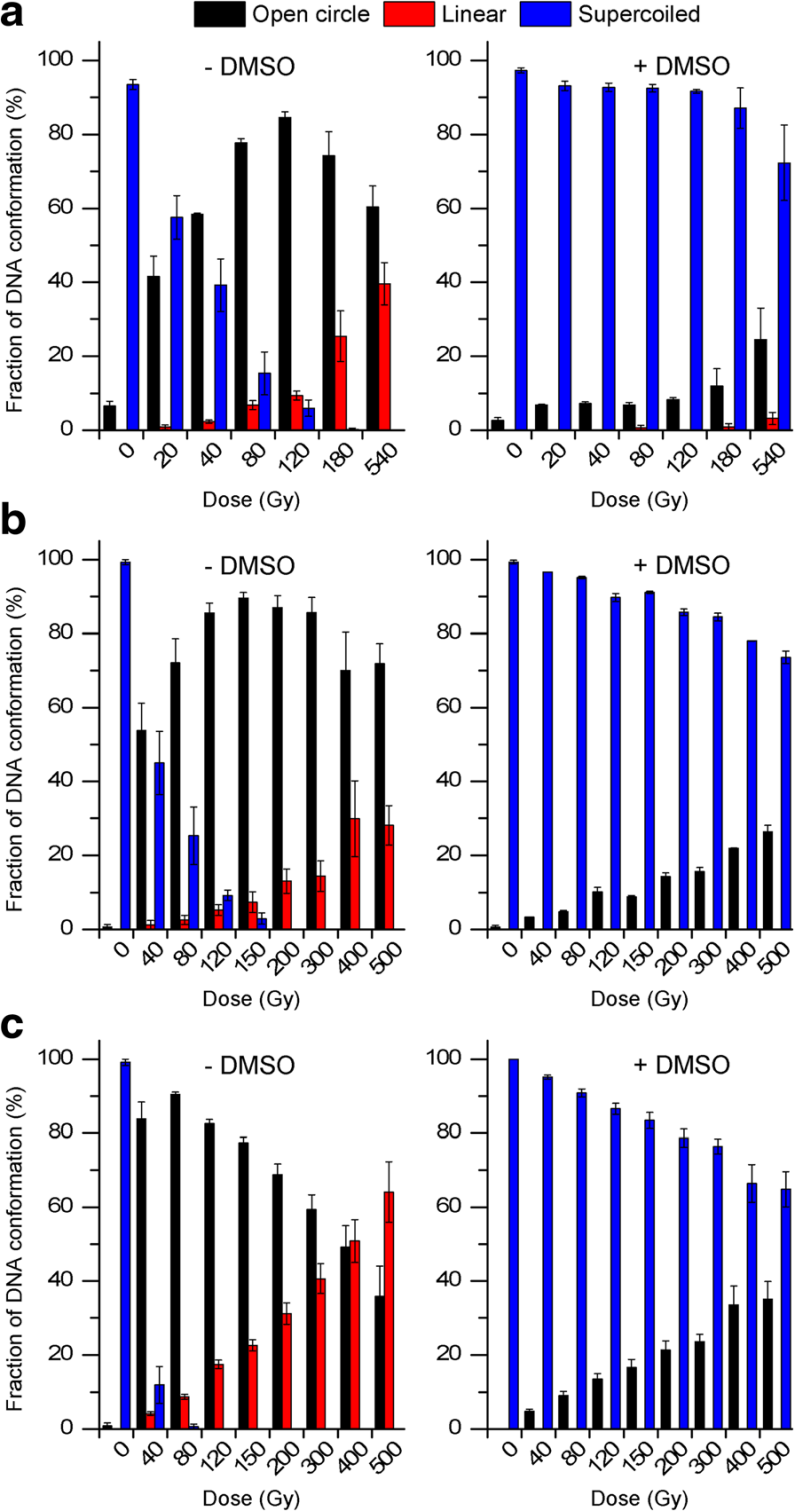
Dose response of ^223^Ra (**a**), ^188^Re (**b**), and ^99m^Tc (**c**) in the absence or presence of DMSO. Dose-dependent increase in open circular DNA and linear DNA was accompanied by the loss of supercoiled DNA. The influence of DMSO on yields of DNA conformational changes shows prevention of SSB and DSB. *Error bars* present the standard errors of the means from three independent experiments

Gel electrophoresis of the plasmid DNA exposed to ^223^Ra, ^99m^Tc, and ^188^Re exhibited a dose-dependent increase in open circular DNA with the appearance of linear DNA at dose points of approximately 20 Gy for ^223^Ra and 40 Gy for both ^188^Re and ^99m^Tc (see Additional file 1: Figure S1). The clear increase in the open circular plasmid forms was up to 120, 150, and 80 Gy for ^223^Ra, ^188^Re, and ^99m^Tc, respectively, which was followed by completely disappearing of the supercoiled forms. Figure 1 shows that at the dose point of 120 Gy for ^223^Ra, ^188^Re and ^99m^Tc irradiation lead to similar yields of OC DNA of approximately 80 %. Exposure to 540 Gy ^223^Ra as well as 500 Gy ^188^Re and ^99m^Tc induced 40, 28, and 64 % linear plasmid conformations, respectively.

To assess the influence of ^•^OH radicals to the formation of SSB and DSB a second set of experiments were carried out in the presence of 0.2 M DMSO. The addition of DMSO had clear effects on the disappearance of the open circular and linear DNA conformations. Nevertheless at the highest dose points significant amounts of OC DNA remained intact after exposure to each of the radionuclides. Notably, only ^223^Ra induced a minor proportion of linear DNA forms that were not prevented by DMSO.

**Fig. 2:**
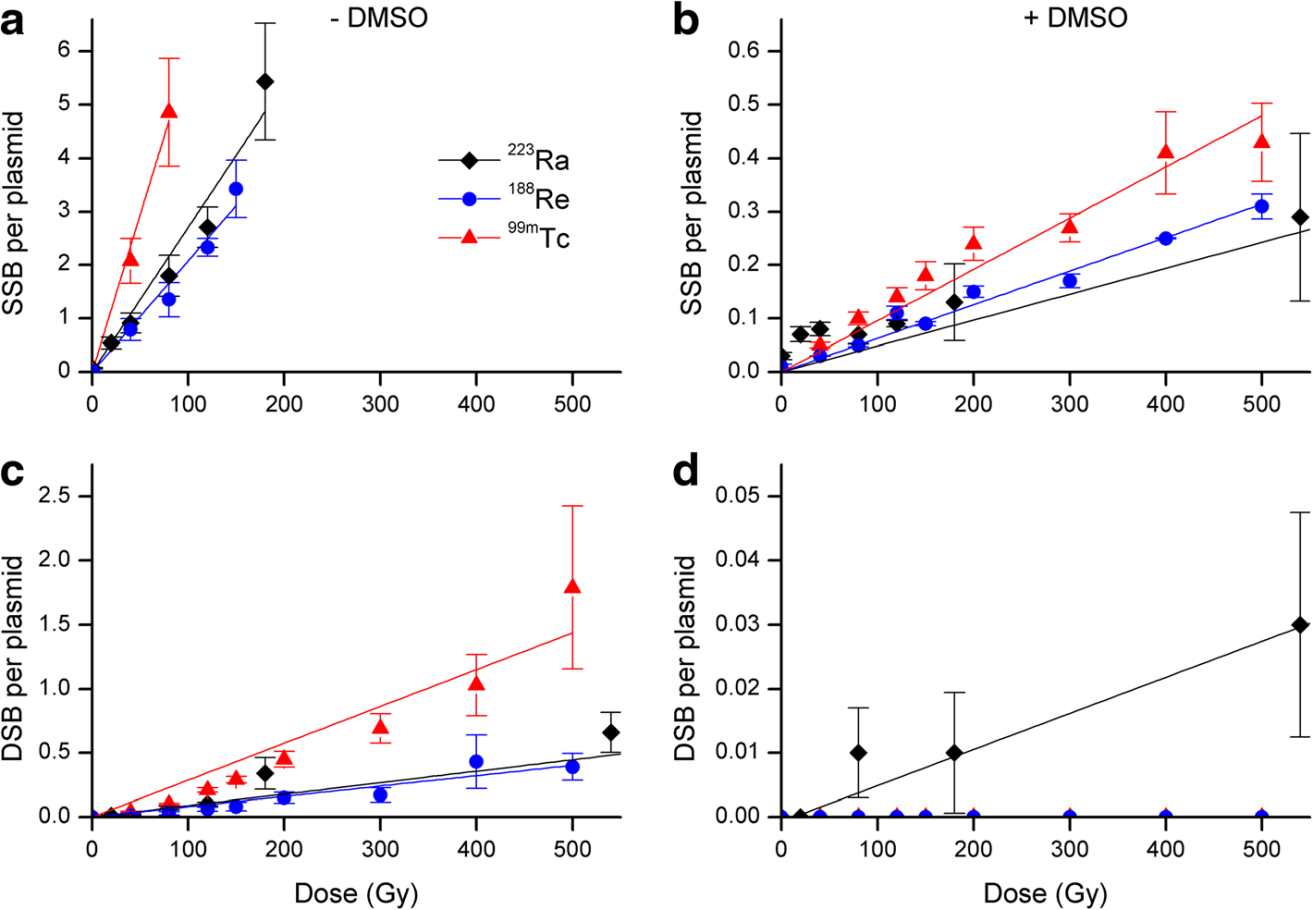
Calculated numbers of SSB (**a**, **b**) and DSB (**c**, **d**) per plasmid molecule in the absence (**a**, **c**) and presence (**b**, **d**) of 0.2 M DMSO for ^223^Ra, ^188^Re, and ^99m^Tc. The mean numbers of SSB and DSB were calculated based on the fluorescence intensities shown in Fig. 1. Panel **a** shows only dose points up to 180 Gy because after higher doses, the SC plasmid forms disappeared. *Error bars* were calculated by the propagation of uncertainty of the standard error of the mean

The mean numbers of SSB and DSB induced by radionuclide exposure were calculated based on the measured fluorescence intensities of plasmid DNA conformations as shown in Fig. 1. With regard to the findings presented in Fig. 2, at the dose point of 80 Gy, ^99m^Tc induced the highest level of SSB (4.9) per plasmid molecule in comparison to ^223^Ra (1.8) and ^188^Re (1.4). At higher doses, the supercoiled plasmid forms disappeared at 180, 150 and 80 Gy for ^223^Ra, ^188^Re, and ^99m^Tc, respectively. Because the calculation of SSB is based on the presence of L and SC plasmid conformations as described in the methods section by *N*_SSB_ = Ln((1−*L*)/SC), Fig. 2a shows only dose points up to 180 Gy. The prevention of SSB by DMSO occurs in a dose-dependent manner for each of the radionuclide with SSB that could not be prevented by DMSO. In accordance to the results presented in Fig. 1, the number of DSB per plasmid molecule after ^99m^Tc exposure was completely prevented by DMSO. In contrast, for ^223^Ra, a minor extent of linear plasmid DNA was preserved.

**Fig. 3:**
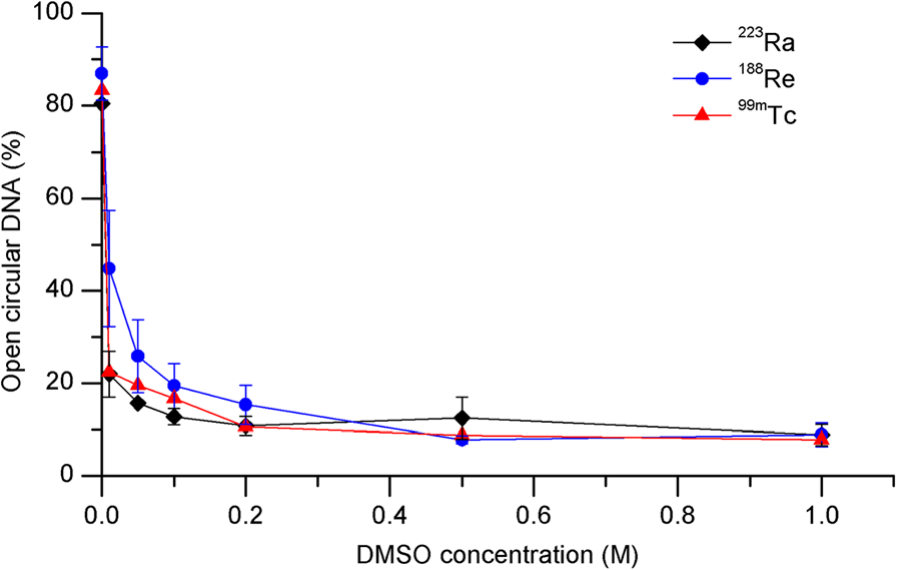
Effect of the DMSO concentrations on open circular fractions of DNA (SSB). Plasmid samples were irradiated with 200 Gy ^223^Ra, ^188^Re, and ^99m^Tc. The relative amount of SSB was determined at various DMSO concentrations. *Error bars* indicate the standard error of the mean from three independent experiments

To test the radical scavenging capacity of DMSO, plasmid DNA was exposed to constant doses of 100, 200, or 400 Gy of each radionuclide in the presence of different concentrations of DMSO (0.05–1.0 M DMSO). Choosing of a common dose for each nuclide was based on the presumption that equal energy doses of the radionuclides lead to equal amounts of ^•^OH radicals. Because all experiments indicated similar curve shapes both regarding the dose as well as the radionuclide, we selected one dose for a clear demonstration of the results. Figure 3 indicates representative findings for 200 Gy and various DMSO concentrations. Increasing the DMSO concentrations resulted in a decrease in open circular DNA (SSB). At nearly 0.2 M DMSO, the curves reached similar plateaus for ^223^Ra, ^188^Re, and ^99m^Tc at 10 % of open circular DNA.

### PC Cl3 cells

**Fig. 4:**
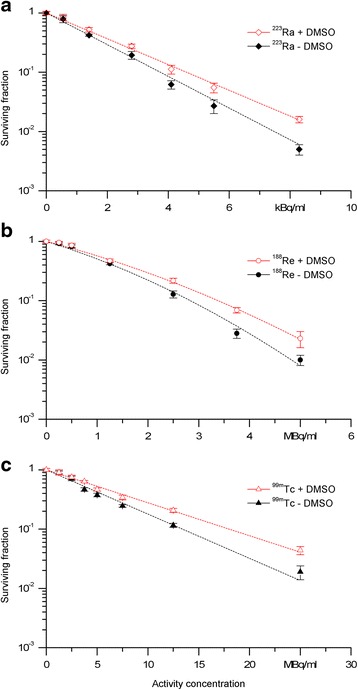
Survival of PC Cl3 cells after exposure to ^223^Ra (**a**), ^188^Re (**b**), and ^99m^Tc (**c**) in the presence or absence of DMSO (0.2 M). The amount of radioactivity was adjusted to reduce survival to about 1 % in the absence of DMSO. The curves were fitted to linear regression. *Error bars* are standard deviations from three independent experiments

Rat thyroid cells were exposed to α-, β-, and Auger electron irradiation aiming first to detect the cellular responses to radiation damage and second to evaluate radioprotection when the cells were preincubated with 0.2 M DMSO. As shown in Fig. 4 treatment with either ^223^Ra, ^188^Re, or ^99m^Tc without DMSO preincubation led to reduced survival fractions in comparison to treatment with DMSO.

In case of ^188^Re calculation, the linear and quadratic parameters and corresponding to the dose response was estimated. For ^223^Ra and ^99m^Tc, the quadratic term of the model was omitted because it did not significantly improve the model adjustment (linear regression, *p* ≥ 0.05). Significant differences between the curves with and without DMSO were determined for each of the radionuclides (linear regression, *p* < 0.001). The appropriate PF values regarding to the regression analysis for ^223^Ra, ^188^Re, and ^99m^Tc are 1.23 ± 0.04, 1.20 ± 0.19, and 1.34 ± 0.05, respectively (see Table 1). The errors were calculated by the propagation of uncertainty. It has to be noted that in case of ^223^Ra and ^99m^Tc (linear curve fit), the $a$ parameters were equivalent to the inverse of A_37_, the activity concentration required to reduce cell survival to 37 %.

**Table 1:** Regression parameters and protection factors obtained by analyzing the survival curves for ^223^Ra, ^188^Re, or ^99m^Tc in the presence or absence of 0.2 M DMSO

Radionuclide	Experimental condition	Regression parameters (mean ± standard error)		Protection factors
		*a*	*b*	
^223^Ra	Without DMSO	0.616 ± 0.012 (kBq/ml)^−1^		1.23 ± 0.04
	With DMSO	0.501 ± 0.008 (kBq/ml)^−1^		
^188^Re	Without DMSO	0.612 ± 0.038 (MBq/ml)^−1^	0.071 ± 0.100 (MBq/ml)^−2^	1.20 ± 0.19
	With DMSO	0.512 ± 0.049 (MBq/ml)^−1^	0.050 ± 0.012 MBq/ml)^−2^	
^99m^Tc	Without DMSO	0.172 ± 0.003 (MBq/ml)^−1^		1.34 ± 0.05
	With DMSO	0.128 ± 0.002 (MBq/ml)^−1^		

**Fig. 5:**
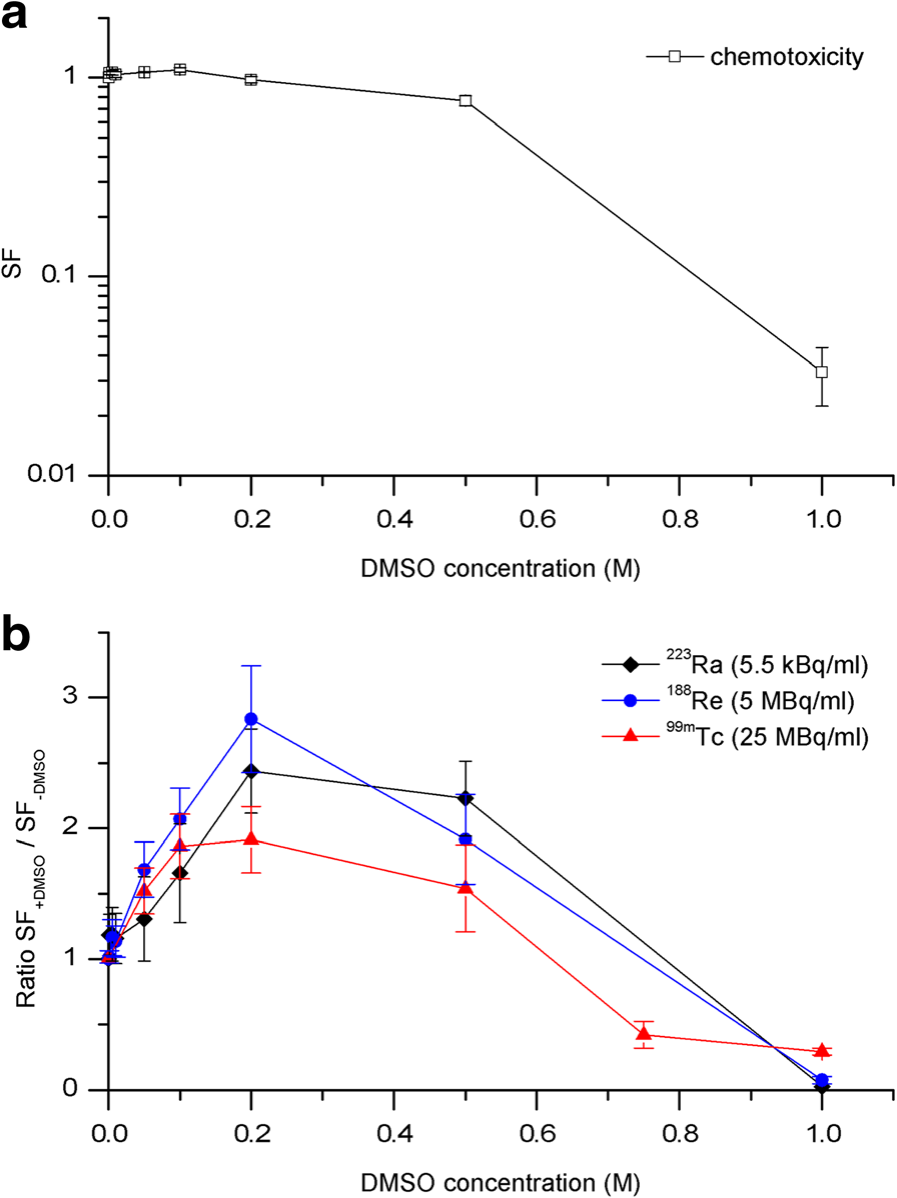
Cytotoxic and radioprotective effects of DMSO. PC Cl3 cells were subjected to different DMSO concentrations (**a**) or irradiated with ^223^Ra, ^188^Re, or ^99m^Tc in combination with DMSO (**b**). The ratio of surviving fractions SF_+DMSO_/SF_−DMSO_ for the radionuclides are shown. *Error bars* are the standard error of the mean from three independent experiments

The chemotoxicity of DMSO as a function of its concentration was also determined (Fig. 5a). Damaging effects on the PC Cl3 cells could be excluded for concentrations ≤0.2 M DMSO. Figure 5b presents the degree of protection when cells were subjected to the radionuclides and treated in combination with various concentrations of DMSO. The activity concentration of each radiation quality was chosen according to their cell killing potential verified by the survival curves (Fig. 4). The appropriate activities correspond to SF_Ra−223_ = 0.027; SF_Re-188_ = 0.01; SF_Tc-99m_ = 0.019.

The degree of protection was calculated by comparing the survival fractions resulting from the combination of the radionuclides and the different DMSO concentrations with the survival fractions following incubation with the radionuclide solution alone. Similar results were analyzed for ^223^Ra, ^188^Re, and ^99m^Tc, and the ratio for protection was found to be in the range of 2–3 at 0.2 M DMSO.

## Discussion

It was generally accepted that high-LET emitters predominantly interact with DNA by direct effects in which the target itself is ionized by the particle rather than by indirect effects of radicals [2]. Hence, it could be expected that the α-emitter ^223^Ra (maximum LET 269 keV/μm) may induce predominantly a direct radiation response in contrast to the β- and Auger electron emitter. The radical scavenger DMSO can be used to protect DNA from sOH radicals that were generated by radiation [28].

### Plasmid DNA-dose response and radioprotection by 0.2 M DMSO

In the present work, plasmid DNA was used as a valuable tool to understand the radiation effects of radionuclides with different LET.

The quantification of DNA damage in plasmid DNA exposed to ^223^Ra, ^188^Re, and ^99m^Tc revealed a dose-dependent increase in open circular and linear DNA conformations. The corresponding yields of SSB and DSB were markedly decreased by the addition of 0.2 M DMSO, with a similar efficiency for each radiation type (Figs. 1 and 2). The modulation of SSB formation resulted in SSB maintenance not preventable in the presence of 0.2 M DMSO, particularly for ^99m^Tc. Probably, SSB might also be caused by direct interactions from the low energy electrons from ^99m^Tc even at high doses up to 500 Gy. Interestingly, ^99m^Tc induced the largest extent of DSB in comparison to ^223^Ra and ^188^Re; however, these DSB could completely inhibited by DMSO. In contrast to ^188^Re and ^99m^Tc, after ^223^Ra treatment, a minor extent of DSB was preserved despite DMSO presence. This finding indicates at least partly a contribution of direct effects from the α-emitter. Ushigome et al. [5] found increasing levels of DSB with an increasing LET of ion-beam irradiations in plasmid DNA films. They reported ratios of SSB to DSB from 12 for helium ions (2.2 keV/μm) to the ratio of 4 for the α-emitter ^141^Pu (148 kev/μm). Our findings did not indicate a clear dependence between DSB induction and LET of the radiation sources. However, when considering the ratios of SSB to DSB at 80 Gy in our study, these values show SSB to DSB ratios of approximately 50 (4.9/0.1; 1.35/0.03) per plasmid molecule for ^99m^Tc and ^188^Re, in contrast to the SSB:DSB ratio for ^223^Ra, which is 24 (1.8/0.07, values from Fig. 2). This difference in the SSB:DSB ratios between ^99m^Tc and ^188^Re in contrast to ^223^Ra may be caused by a higher contribution of direct radiation effects from the α-emitter ^223^Ra. Peak et al. analyzed SSB and DSB yields induced by both a γ-emitter and high-LET neutrons. They supposed that the SSB and DSB caused by the neutrons are, to some extent, due to DNA breaks initiated by indirect effects [2]. This is partly consistent with our results, which indicated for the α-emitter ^223^Ra, a reduction of SSB and DSB in the presence of DMSO, suggesting also indirect effects that are mediated by ^•^OH radicals. Our group recently described the protective effect of DMSO against SSB and DSB formation by ^99m^Tc-HYNIC-DAPI. We demonstrated in that previous study a negligible decrease of the SSB and DSB numbers in the presence of 0.2 M DMSO, inferring mainly a direct interaction of ^99m^Tc-HYNIC-DAPI with DNA [29]. Yet, as shown in the present work, the α-emitter ^223^Ra induced small amounts of linear DNA fractions (DSB) that were maintained despite DMSO modulation (Fig. 2). This observation supports the assumption that direct effects from α-particles are at least partly involved in DNA damage.

### Plasmid DNA—variation of DMSO concentration

The open circular DNA forms (SSB) that remain intact after application of high dose levels (>200 Gy) of each radiation type (Fig. 1) may suggest a saturation of the radical scavenger capacity. To verify this hypothesis, we performed experiments with varying DMSO concentrations (0.05–1.0 M DMSO) while keeping the radioactivity concentrations constant. We applied doses of 200 Gy of each radionuclide, choosing the maximal level of the SSB extent. Even small DMSO concentrations cause a steep decline in the open circular DNA values, reaching a similar plateau at 0.2 M DMSO for ^223^Ra, ^188^Re, and ^99m^Tc (Fig. 3). This means that the radical scavenging capacity is dependent on the number of DMSO molecules and radioactivity concentrations. Unexpectedly, we observed a residual level of SSB although the DMSO concentration had increased. One reason for this might be the contribution of secondary radicals (^•^CH_3_), which are also able to react with DNA but to a minor extent [28]. Further, the formation of hydrogen radicals (^•^H) or solvated electrons (e^−^_aq_) probably achieved maintenance of SSB because DMSO is primarily able to capture hydroxyl radicals. Our observations can probably be ascribed to direct effects that enable the induction of direct SSB.

### PC Cl3 cells—dose response in absence and presence of 0.2 M DMSO

A variety of studies aimed to detect radiation effects in mammalian cells using different radical scavengers, primarily DMSO [9, 12, 14]. We used the standard colony formation assay to estimate the clonogenic cell survival. The PC Cl3 cells are characterized by expression of the sodium iodide symporter (NIS) which allows the accumulation of ^188^Re and ^99m^Tc into the cells. For precise dose calculations, data on subcellular radionuclide distribution are required [30]. Because our study focused on the influence of DMSO for each radionuclide but not to compare the radiotoxicity between them, such experiments were not performed.

Exposing PC Cl3 cells to each of the radionuclides in the absence and presence of 0.2 M DMSO leads to comparatively low but statistically significant differences between the SF assessed in the presence or absence of DMSO. The protection factors were found to be in the same range for ^223^Ra, ^188^Re, and ^99m^Tc (1.23 ± 0.04, 1.20 ± 0.19, and 1.34 ± 0.05, respectively). Besides, the calculation of the statistical error suggests no different protection effects among the radionuclides. However, the highest factor was calculated for ^99m^Tc. An explanation for this may be the higher contribution of radical mediated radiation effects.

Bishayee et al. [12] determined the influence of 1.28 M DMSO on the survival of cells exposed to ^210^Po, ^131^IIdU, ^125^I-IdU, and ^3^H_2_O. They calculated dose-modifying factors (DMF) that were given by the ratio of the values *A*_0_ in the presence and absence of DMSO in a similar manner to the protection factors calculated for our findings. The highest protection obtained by this group was for β-particles of ^3^H_2_O (DMF = 2.9), and the protection efficiencies for intracellular ^210^Po, ^131^I-IdU, and ^125^I-IdU were 0.95, 2.3, and 2.6, respectively. When comparing the protection factors from Bishayee et al., it appears that the values for β- and Auger emitters were higher (2.3, 2.6, 2.9) than those resulting from our observations (1.20, 1.34). No protection against high-LET α-particles from ^210^Po (DMF = 0.95) was shown in contrast to our experiments with ^223^Ra (PF = 1.23). However, these authors used a much higher DMSO concentration and maintained the cells at 10.5 °C, which is obviously different to our experimental setup (37 °C). The varying experimental setups may produce different results.

In addition to this, our results are consistent with a study reported by deLara et al. [9] showing that protection by 0.1 M DMSO against α-particles (^238^Pu, PF = 1.14) was slightly less than that for γ-radiation (^60^Co, PF = 1.2).

### PC Cl3 cells—variation of DMSO concentration

The influence of different DMSO concentrations on cell survival was analyzed for constant radioactivity concentrations of ^223^Ra, ^188^Re, and ^99m^Tc. In the presence of DMSO, the cell survival increases as a function of the concentration (Fig. 5b). The highest protection effect was analyzed at 0.2 M DMSO holding true for all radionuclides. At concentrations ≥0.5 M DMSO alone, cytotoxic effects of DMSO influenced the survival. Based on the reported studies [9, 12, 31], for mammalian cells, it could be assumed that radiation-induced damage decreases upon increasing the DMSO concentration. These reports are in agreement with the findings of our study. In fact, the cytotoxic effects of DMSO cannot be entirely excluded, due to length of time for which cells are irradiated with radionuclides.

### Comparison of the effects in plasmid DNA and PC Cl3 cells

Regarding the two biological systems, we found that DMSO could diminish DNA damage in a similar manner for each of the radiation sources, as shown in Figs. 1 and 4. In plasmids, the protective effect of DMSO leads to considerably differences between the extent of DNA damage (SSB, DSB) as well as the inhibition of DSB was more pronounced with ^99m^Tc in comparison to ^223^Ra. As presented in Fig. 2, the DSB from ^99m^Tc were completely inhibited under radical scavenger conditions unlike to the DSB from ^223^Ra. Together, plasmid experiments indicated at least to a minor extent a relationship between the properties of the radiation quality and the type of DNA damage. Analyzing the cell survival curves, we calculated protection factors between 1.20 and 1.34. In contrast to the plasmids, in PC Cl3 cells, we found only slightly differences when comparing the survival fractions, measured in the absence or presence of DMSO. However, the statistical evaluation revealed the significance of the DMSO protection for each of the radionuclides. Generally, there are distinct differences in how the biological systems respond to radiation. A variety of well-defined repair enzymes and peptides exist in cells that are able to eliminate the background level of free radicals. Since our experiments were conducted at physiological temperatures for the cells (37 ° C), the existence of the background level of radical eliminating enzymes should be considered when comparing the extent of DMSO protection in plasmids and cells. Further, the differences between the two biological systems are caused by the different topological characteristic such as DNA conformation and DNA environment. Plasmid DNA is chromatin free and non-compacted, and thus, radionuclides, radicals, and radical scavenger are able to compete with plasmid DNA without any biological barriers. In the naked DNA of plasmids, there are large distances separating the DNA loops; the decays can only produce DSB or SSB in the case of a high hit probability of the radiation. That is quite different with cells where DNA is tightly packed enabling damage by decaying radionuclides to adjacent DNA sites. When comparing DNA damage induced in plasmids with those in cells, the biological background mentioned above seems to influence the radiobiological effects.

## Conclusions

Dose-dependent radiation effects were found for high-LET emitter as well as for low-LET emitter, applicable for plasmid DNA and cells. We detected only minor relationships between the physical characteristic of the radionuclides and the biological response of plasmid DNA and cells. All together, the radioprotective action of DMSO was in the same range for the α-particles and electrons assuming that indirect radiation effects by radicals are dominant for the mechanism of DNA damage and cell inactivation. With respect to a therapeutic use, our findings may contribute to fundamental knowledge about the mechanistic basis of α-particle-induced DNA damage and cell inactivation.

## Abbreviations

ATCC, American Type Culture Collection; DSB, double strand break; DMSO, dimethyl sulfoxide; EDTA, ethylenediaminetetraacetic acid; Gy, Gray; h, hour; LET, linear energy transfer; eV, electron volt; pUC19, plasmid University of California 19; SSB, single strand break

## Additional file

Additional file 1: Representative agarose gels of ^223^Ra, ^188^Re, and ^99m^Tc in the absence (lanes 1–6) and presence (lanes 7–12) of 0.2 M DMSO. Plasmid DNA was treated with 20, 40, 80, 120, 180, 540 Gy of either ^223^Ra (A); 40, 80, 120, 150, 200, 500 Gy ^188^Re (B) or ^99m^Tc (C), respectively. Lanes C are the control plasmid DNA without irradiations. (PPTX 191 kb)
