# Supplementary material for: The effect of dimethyl sulfoxide on the induction of DNA strand breaks in plasmid DNA and colony formation of PC Cl3 mammalian cells by alpha-, beta-, and Auger electron emitters 223Ra, 188Re, and 99mTc
Source: EJNMMI Res. 2016 Jun 3;6:48. doi: 10.1186/s13550-016-0203-x (PMC4893047; doi:10.1186/s13550-016-0203-x)
Supplement: Additional file 1: — Representative agarose gels of 223Ra, 188Re, and 99mTc in the absence (lanes 1–6) and presence (lanes 7–12) of 0.2 M DMSO. Plasmid DNA was treated with 20, 40, 80, 120, 180, 540 Gy of either 223Ra (A); 40, 80, 120, 150, 200, 500 Gy 188Re (B) or 99mTc (C), respectively. Lanes C are the control plasmid DNA without irradiations. (PPTX 191 kb) [file 13550_2016_203_MOESM1_ESM.pptx]

## Slide 1
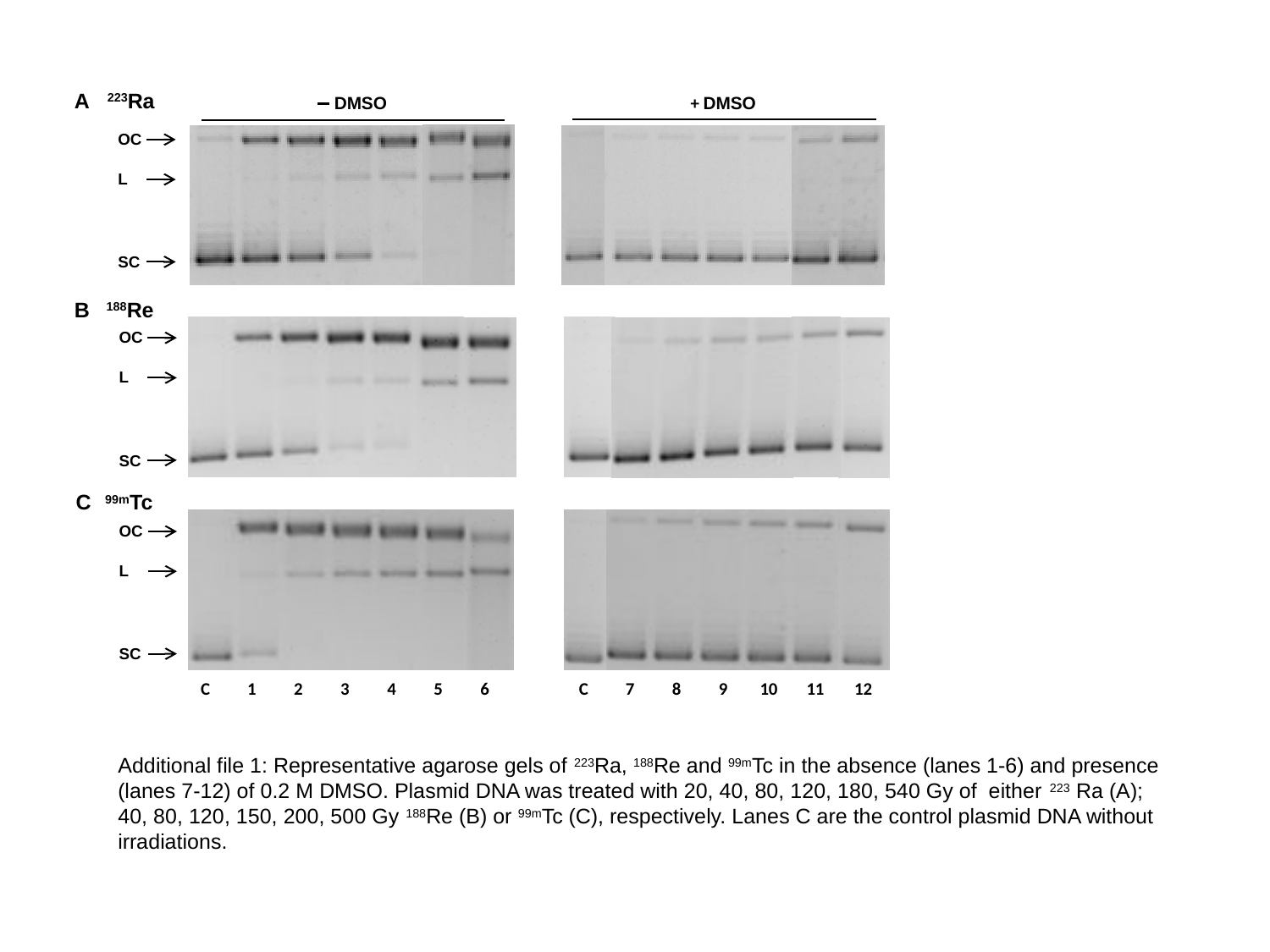

A
223Ra
+ DMSO
­­ DMSO
OC
L
SC
B
188Re
OC
L
SC
C
99mTc
OC
L
SC
C
7
8
9
10
11
12
C
1
2
3
4
5
6
Additional file 1: Representative agarose gels of 223Ra, 188Re and 99mTc in the absence (lanes 1-6) and presence (lanes 7-12) of 0.2 M DMSO. Plasmid DNA was treated with 20, 40, 80, 120, 180, 540 Gy of either 223 Ra (A); 40, 80, 120, 150, 200, 500 Gy 188Re (B) or 99mTc (C), respectively. Lanes C are the control plasmid DNA without irradiations.
